# Direct fluorescent labelling of clones by DOP PCR

**DOI:** 10.1186/1755-8166-1-3

**Published:** 2008-03-26

**Authors:** Liesbeth Backx, Reinhilde Thoelen, Hilde Van Esch, Joris R Vermeesch

**Affiliations:** 1 Center for Human Genetics, University Hospital Leuven, Herestraat 49, B-3000 Leuven, Belgium

## Abstract

**Background:**

Array Comparative Genomic Hybridisation (array CGH) is a powerful technique for the analysis of constitutional chromosomal anomalies. Chromosomal duplications or deletions detected by array CGH need subsequently to be validated by other methods. One method of validation is Fluorescence *in situ *Hybridisation (FISH). Traditionally, fluorophores or hapten labelling is performed by nick translation or random prime labelling of purified Bacterial Artificial Chromosome (BAC) products. However, since the array targets have been generated from Degenerate Oligonucleotide Primed (DOP) amplified BAC clones, we aimed to use these DOP amplified BAC clones as the basis of an automated FISH labelling protocol. Unfortunately, labelling of DOP amplified BAC clones by traditional labelling methods resulted in high levels of background.

**Results:**

We designed an improved labelling method, by means of degenerate oligonucleotides that resulted in optimal FISH probes with low background.

**Conclusion:**

We generated an improved labelling method for FISH which enables the rapid generation of FISH probes without the need for isolating BAC DNA. We labelled about 900 clones with this method with a success rate of 97%.

## Background

Fluorescence *in situ *hybridisation (FISH) is a widely used technique for visualization and analysis of chromosomal anomalies [1,2]. Basic requirements are specific DNA probes in sufficient amounts as well as a labelling protocol with good reproducibility. Traditionally, fluorophores or hapten labelling is performed by nick translation or random prime labelling of purified BAC products. Other methods for labelling are primer extension and chemical coupling (by means of amino-group modified dNTPs) and also a number of other technologies [1-3]. These are all labour intensive procedures including culturing, isolating and labelling of the BAC DNA. However, since for array CGH, targets (DNA spotted on the array) have been generated from degenerate oligonucleotide primed (DOP) amplified BAC clones, we aimed to use these DOP amplified BAC clones as the basis of an automated FISH labelling protocol [4-7]. Unfortunately, the labelling of DOP amplified BAC clones by means of either traditional labelling methods or a PCR reaction with the DOP 4 primer (see below) resulted in high levels of background. We hypothesised that this background may be caused by the formation of panhandle structures of the single-stranded PCR fragments due to intramolecular re-annealing of the primers. During this re-annealing process, multiple fragments could be trapped and they formed larger networks of fluorescent labelled molecules causing high background signals. We reasoned that small degenerate oligonucleotide primers would not be able to form such panhandles. Therefore we set out a PCR with degenerate oligonucleotides, of which only the 5 prime end ten nucleotides are complementary to the template and developed an efficient labelling technique.

## Results

**Figure 1:**
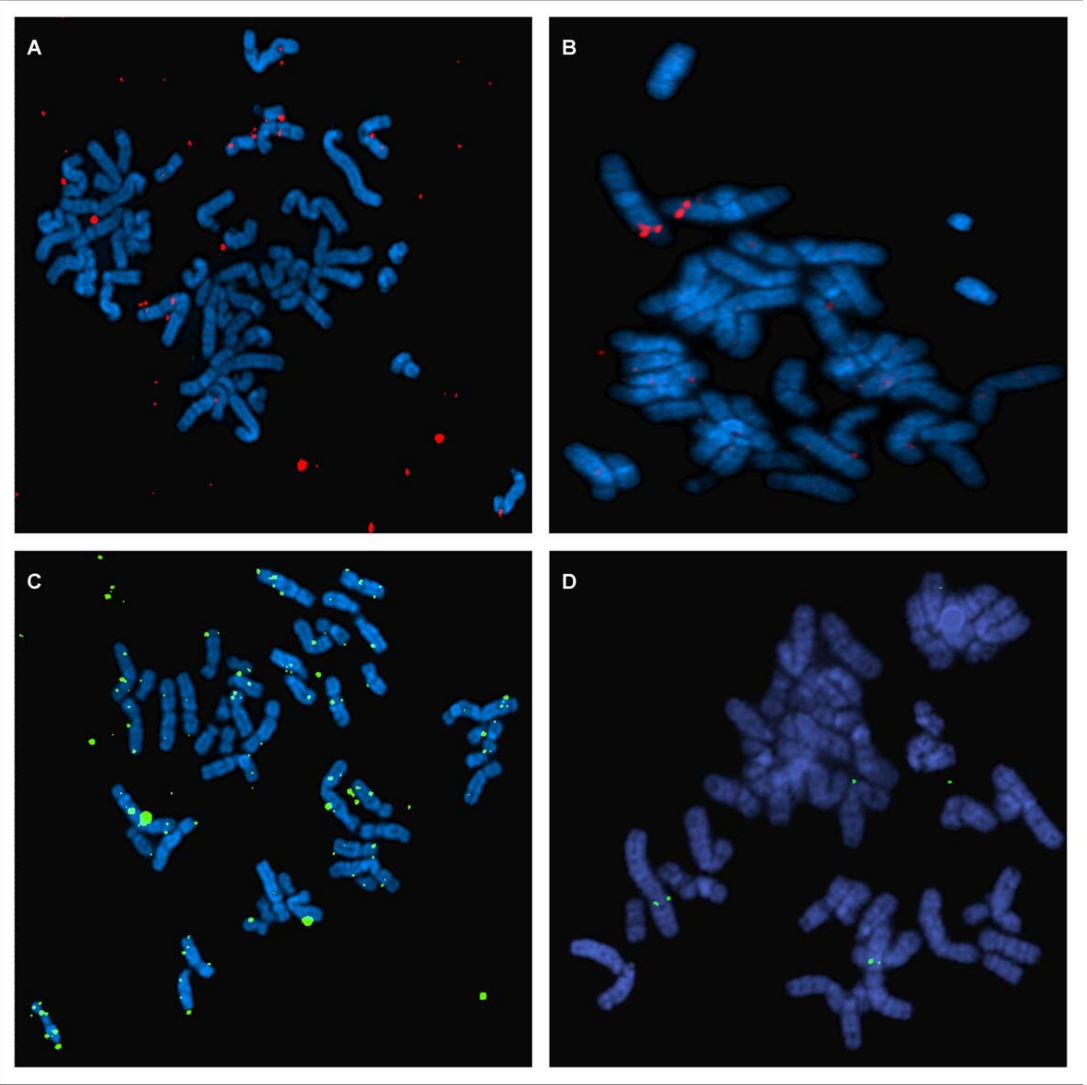
**Results of FISH**. A: DOP amplified BAC clone (BAC RP11-393B19) labelled with SpectrumOrange™- dUTP by means of the DOP4 primer in a PCR amplification reaction; B: DOP amplified BAC clone (BAC RP11-393B19) labelled with SpectrumOrange™- dUTP with the optimized protocol; C: DOP amplified BAC clone (BAC RP11-303I4) labelled with biotin d-CTP using the DOP4 primer in a PCR amplification reaction.; D: DOP amplified BAC clone (BAC RP11-303I4) labelled with biotin d-CTP with the optimized protocol.

DOP amplified BAC DNA was labelled by PCR using either the DOP 4 primer or a mix of DOP 1, 2 and 3 primers. FISH with the labelled amplified BAC DNA by means of the DOP 4 primer showed high background signals (Figure 1 A,C). FISH with the labelled amplified BAC DNA by means of the mix of DOP 1, 2 and 3 primers resulted in optimal FISH probes with lower background and provided a good signal to noise (Figure 1 B,D).

We also compared different fluorophores or hapten for PCR incorporation in the DOP amplified BAC clones including SpectrumGreen™- dUTP, SpectrumOrange™- dUTP or biotin-dCTP. FISH signals were obtained with all 3 of them, but the BAC clones labelled with SpectrumOrange™- dUTP provided the best hybridisation signals (see Figure 1B). The BAC clones labelled with SpectrumGreen™- dUTP or biotin-dCTP showed FISH signals of lower intensity (see Figure 1D).

## Discussion and conclusion

Array CGH is becoming a valuable and powerful technique for the detection of chromosomal aberrations. These aberrations subsequently need to be validated by other methods. One method of validation is FISH, which is a labour intensive method [1,8]. We set out to use the DOP amplified BAC clones, generated for the array target, as the basis of an automated FISH labelling protocol and showed that amplified BAC DNA labelled with our optimized DOP PCR protocol provides good hybridisation signals and lower background. This labelling method enables the rapid generation of FISH probes without the need for isolating BAC DNA from bacterial cultures. Not only BAC, but also cosmid and fosmid clones can be used as a substrate for labelling (data not shown).

Regarding the choice of fluorophores, the use of biotin-dCTP requires signal amplification after hybridisation, which is more time consuming than labelling with specific fluorophores, like SpectrumGreen™- dUTP, SpectrumOrange™-dUTP, which are pre-labelled and do not require signal amplification after hybridisation. Thus far, we have labelled about 900 clones with this method using SpectrumOrange™-dUTP with a success rate of 97%.

FISH enables the detection of the location of the aberration on the chromosome and is the most suitable technique to validate deletions. Furthermore, FISH enables detection of translocations and reveals more complex rearrangements not detected by array CGH. However, FISH is not suitable for detection of tandem duplications or deletions that are smaller in size than the probe used. This is a limitation for validation of small imbalances detected by higher resolution arrays. In these cases, molecular methods that can combine a number of investigated loci in a single reaction are preferable, such as Multiplex Ligation-dependent Probe Amplification (MLPA) and quantitative PCR.

In conclusion, we present a fast and accurate FISH labelling method.

## Methods

The following primers were used for DOP-PCR amplification as described previously [4]: DOP1: 5'-CCGACTCGAGNNNNNNCTAGAA-3', DOP2: 5'-CCGACTCGAGNNNNNNTAGGAG-3', DOP3: 5'-CCGACTCGAGNNNNNNTTCTAG-3' and DOP4: 5'-GGAAACAGCCCGACTCGAG-3' (Eurogentec, Seraing, Belgium).

The target DNA for array CGH was isolated and purified as previously described [8]. This BAC DNA was preamplified by PCR by means of a DOP 1, 2, 3 primermix followed by a second amplification round with an aminolinked primer (5'-NH2-GGAAACAGCCCGACTCGAG-3') [4,8,9]. An aliquot, 10 μl, of these DOP PCR products were diluted 10 times and stored at -20°C in 96 well plate and were then ready for use as a template in the labelling reaction.

Subsequently, the DOP amplified BAC DNA was labelled as follows: in a total volume of 50 μl containing 5 μl of 15 μM DOP 1, 2, 3 primermix, 5 μl of 10× PCR buffer without MgCl_2_, which is specially designed for use with Platinum^® ^*Taq *DNA polymerase (Invitrogen, Carlsbad, CA) and 5 μl 50 mM MgCl_2_. For the dNTP's we used 1 μl 10 mM dATP, dCTP, dGTP each, 0.7 μl 10 mM dTTP, 1 μl of 1 mM SpectrumGreen™-, or SpectrumOrange™- dUTP (Abbott laboratories, IL) or 5 μl 10× dNTP mixture containing 1 mM biotin-14-dCTP, 1 mM dCTP, 2 mM dATP, 2 mM dGTP, 2 mM dTTP in 10 mM Tris-HCl (pH 7.5), 1 mM Na2 EDTA (Bioprime DNA labelling system, Invitrogen, Carlsbad, CA). As DNA polymerase we used 0.5 μl Platinum^® ^*Taq *DNA polymerase (Invitrogen, Carlsbad, CA) and finally added H_2_O to 48 μl and 2 μl of the DOP amplified BAC DNA. All PCR reactions were perfomed on a thermocycler (GeneAmp9700, Applera, Nieuwekerk a/d Ijzer, The Netherlands). After initial denaturation at 95°C for 10 minutes, the reaction was as follows: 35 cycles of 95°C for 1 min, 60°C for 1 min and 72°C for 1 min and a final extension step of 72°C for 10 min.

The reagents, volumes and reaction conditions of our optimized DOP PCR labelling protocol are summarized in Table 1. The quality of the PCR products can be analyzed on a 2% agarose-ethidium-stained gel. A smear of bands from 200 to 2000 basepairs should be visible. Purification of the PCR product was perfomed with the Qiaquick 8 PCR purification kit (Qiagen NV, Venlo, The Netherlands) by means of QIAvac 6S vacuum according to instructions of the suppliers.

**Table 1:** The optimized labelling protocol

**PCR mix**	
**Reagent**	**Volume**

Mix of DOP1,2,3 primers (15 μM)	5 μl
10× PCR Buffer w/o MgCl_2_	5 μl
50 mM MgCl_2_	5 μl
dATP (10 mM)	1 μl
dGTP (10 mM)	1 μl
dCTP (10 mM)	1 μl
dTTP (10 mM)	0.7 μl
x- dUTP (1 mM) *	1 μl
H_2_O	27.8 μl
Platinum Taq DNA Polymerase	0.5 μl
DOP amplified BAC clone	2 μl
Total volume	50 μl

* SpectrumGreen™- dUTP (Vysis), SpectrumOrange™- dUTP (Vysis) or biotin-dCTP

FISH was used to analyse the signal intensity and background levels of the BAC clones labelled by DOP-PCR. Before FISH, cells were air-dried on slides and pretreated with pepsin followed by fixation with a 1% free formaldehyde solution and subsequently dehydrated with ethanol. FISH with biotin as hapten was performed as previously described [10]. After hybridisation overnight at 37°C, the slides were washed in 0.4 × SSC/0.3% NP40 solution at 72°C for one minute, one minute at 2 × SSC/0.1% NP40 solution at RT and one minute at 2× SSC. The cells were counterstained with DAPI and the slides were mounted in Vectashield mounting medium (Vector Laboratories, Burlingame, CA). The signal was visualised by digital imaging microscopy with Cytovision capturing software (Applied Imaging, Santa Clara, CA).

## Abbreviations

DOP PCR: degenerate oligonucleotide-primed PCR; FISH: fluorescence in situ hybridisation; BAC: bacterial artificial chromosome; CGH: comparative genomic hybridisation

## Competing interests

The author(s) declare that they have no competing interests.

## Authors' contributions

LB is a PhD student who performed the experiments for optimizing the protocol. JRV is the head of the cytogenetics laboratory and participated in the design of the protocol. HVE is a clinical geneticist and helped correcting the manuscript. RT is a technician who helped with the interpretation of the FISH results. All authors read and approved the final manuscript.
